# Micro-computed tomography to visualize preserved vascular architecture in decellularized human vaginal tissue: explorative study

**DOI:** 10.1038/s41598-025-14452-8

**Published:** 2025-08-20

**Authors:** Jayson Sueters, Daniel Docter, Freek Groenman, Sophie C. Visser, Bernadette S. de Bakker, Theo H. Smit, Judith A. F. Huirne

**Affiliations:** 1 Department of Gynaecology, Amsterdam UMC – Location VUmc, De Boelelaan 1117, 1105 AZ Amsterdam, The Netherlands; 2 Department of Obstetrics and Gynecology, Amsterdam UMC – Location VUmc, De Boelelaan 1117, 1105 AZ Amsterdam, The Netherlands; 3 AGEM – Amsterdam Gastroenterology Endocrinology Metabolism, Amsterdam UMC – Location AMC, Meibergdreef 9, 1105 AZ Amsterdam, The Netherlands; 4 Department of Obstetrics and Gynecology, Amsterdam UMC – Location University of Amsterdam, Meibergdreef 9, 1105 AZ Amsterdam, The Netherlands; 5 Department of Medical Biology, Amsterdam UMC – Location AMC, Meibergdreef 9, 1105 AZ Amsterdam, The Netherlands; 6 Amsterdam Reproduction and Development Research Institute, Amsterdam UMC, Meibergdreef 9, 1105 AZ Amsterdam, The Netherlands; 7 Centre of Expertise on Gender Dysphoria, Amsterdam UMC – Location VUmc, De Boelelaan 1117, 1105 AZ Amsterdam, The Netherlands

**Keywords:** Decellularized vagina matrix, Neovagina, Micro-computed tomography, Vasculature, Blood vessel density, Health care, Medical research, Engineering

## Abstract

When a vagina is dysfunctional or absent, a neovagina is typically created using an intestinal or dermal graft. This causes complications and complaints from different inherent tissue properties. We recently developed an organ-specific matrix from healthy, human vaginal wall tissue by decellularization, and demonstrated native-like matrix protein composition and mechanical properties. However, the preservation of its vascular structures is unknown, but plays a crucial role for recellularization, tissue survival and graft integration during clinical applicability. Therefore, in this explorative study the vascular architecture of human native and decellularized vagina tissue were assessed and compared. Micro-computed tomography (micro-CT) was used to analyze and quantify the three-dimensional micromorphology of blood vessels within native and decellularized human tissue. The vascular density, connectivity, length and thickness was determined for 11 test volumes from three healthy transgender donors. At a 5 μm isotropic voxel size resolution, quantitative assessment demonstrated no significant changes of the vascular density upon decellularization. However, significant decellularization-induced changes were seen for vascular connectivity (*P* < 0.001), vascular thickness (*P* < 0.01) and vascular length (*P* < 0.001). Micro-CT imaging is an effective strategy for high-resolution analysis of blood vessel structures in human (vaginal) tissue, and in bio-engineered tissue constructs. Despite significant loss of small capillaries, preservation of vascular architecture in chemically decellularized tissue was confirmed for large vascular structures through intra-patient comparison with native tissue. This validates an important aspect and predictor for functionality of a human vagina-specific matrix in terms of graft survival and integration.

**Supplementary Information:**

The online version contains supplementary material available at 10.1038/s41598-025-14452-8.

## Introduction

When a functional vagina is absent, this hampers an individual’s quality of life and psychological well-being^1^. By surgical reconstruction, known as vaginoplasty, a neovagina can be formed with natural-like function and sensation^2–6^. Generally, this involves recreation of the vagina wall using autologous intestinal or dermal grafts, resulting in well-reported limitations, risks and drawbacks^1,6–18^ due to inherently different tissue properties. The difficulty of mimicking the complex vagina especially concerns its physiology for secretion of discharge, and its unique mechanical properties to support pelvic organs while allowing large deformations during vaginal intercourse and childbirth^19^. Hence, human vagina-derived decellularized matrices can provide a valuable resource, as they often contain native ECM structures, composition and mechanical properties^20^. However, for their natural viscoelastic properties^21^, decellularized vaginal matrices (DVMs) rely on complete preservation of the extracellular matrix (ECM) and ECM proteins like collagen and elastin. In our previous study, we constructed a human-derived DVM with adequate mechanical responses but also a seemingly visual reduction of collagen and elastin^20^. Next, to prepare the clinical applicability of human DVM as vagina transplant, (creation of) a vascular system is needed.

**Fig. 1:**
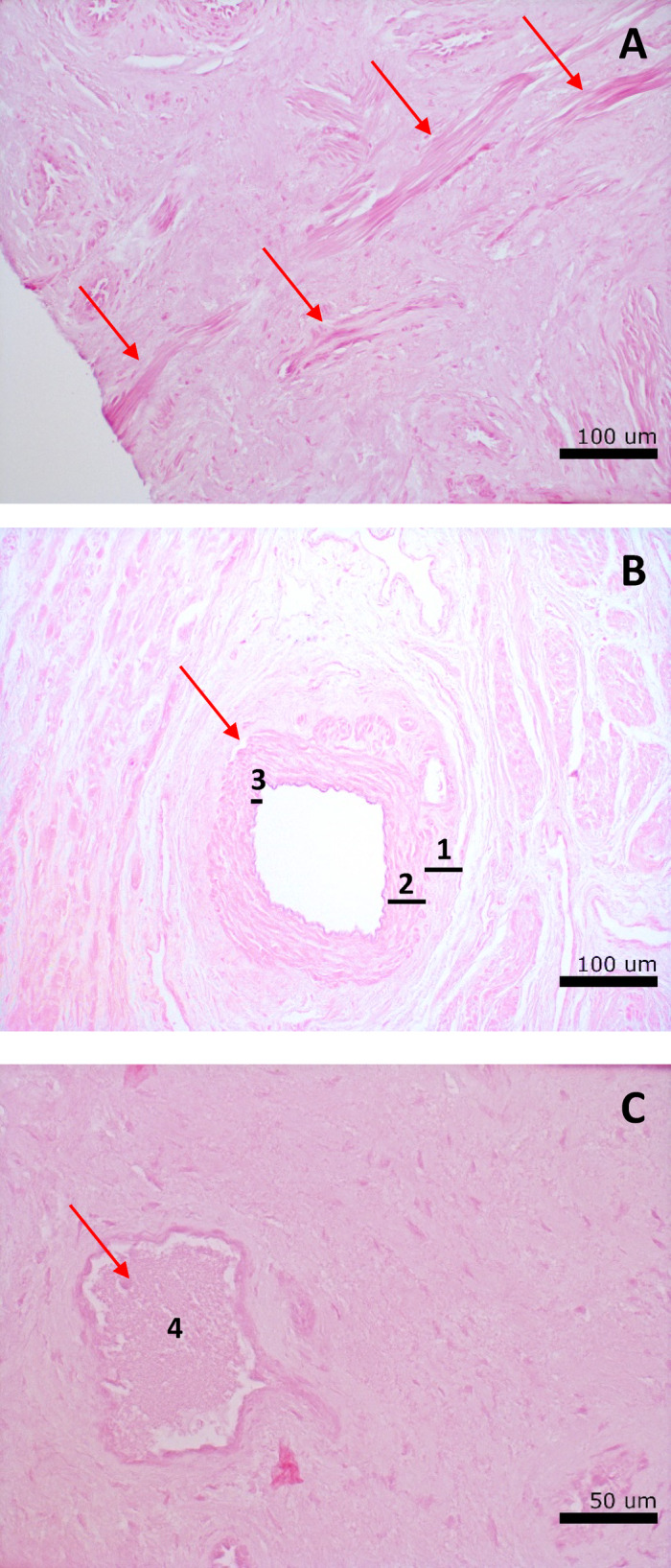
Histology of decellularized human vaginal matrix. histological assessment with haematoxylin- and eosin-staining of decellularized human vaginal tissue depicts vascular structures (indicated by red arrows). (**A**) Endodermal sample illustrates several longitudinal-sections of arteries. (**B**) Mesodermal sample demonstrates cross-section of an artery with clear tunica externa^1^, thick tunica media (muscle layer)^2^ and tunica intima^3^. (**C**) Endodermal sample illustrates cross-section of a vein, with thinner wall and muscle layer compared to artery. Large lumen visible^4^.

In biomaterials, like our DVM, a vasculature allows (in)growth of blood vessels and nerves, matrix recellularization and its survival, especially at sizes approximating the human vaginal anatomy. In addition, angiogenesis and vasculogenesis, together with neurogenesis, are required for successful DVM integration and functionality^22^. An artificial vasculature can be constructed with neo-vascularization by cells or biological factors, 3D- or bio-printing, or micro-perforations with needles, but this might not be necessary. Studies have shown the intact ECM of native vasculature in decellularized liver^23,24^, heart^25,26^ and kidney^27,28^. Furthermore, histological observations of our DVM^20^ demonstrated vascular-resembling features (Fig. 1). However, the vascular wall consists of cells and an ECM architecture with laminin, collagen, elastin and glycosaminoglycans (GAG). With the chemical removal of cells, and potentially that of collagen and elastin, the preservation of a vascular architecture in our DVM is thus uncertain. Furthermore, this preserved vasculature might differ for the Müllerian duct-derived (proximal, mesoderm) and urogenital tract-derived vagina (distal, endoderm)^29^ despite similar native microvessel quantities^30^, as these differ in epithelium layer thickness^31^. Therefore, this study aims to compare the blood vessel density of native vaginal tissue with DVM of mesodermal and endodermal origin from the same individual, as an indicator for preservation of the vascular architecture upon decellularization for DVM survival and integration upon implantation. In broader perspective, we want to determine whether the vascular architecture in DVM is preserved or that a neo-vascularization approach is needed before seeding of the DVM with cells.

To investigate the vasculature, micro-computed tomography (micro-CT or µCT) is especially suitable as it allows 3D imaging with high reproducibility, excellent spatial resolution and high-throughput analysis^32^. Meanwhile, techniques like histology and fluorescence microscopy are limited in penetration depth, their study area or 2D rather than 3D nature^33,34^. Especially for visualization and quantification^32^ of micro-vessels (around 10–50 μm in diameter)^35,36^ that deliver oxygen and nutrients to cells^37^, the 1-µm spatial resolution^34^ of contrast-enhanced micro-CT^38–40^ is valuable. Here, the three-dimensional micromorphology of blood vessels will be visualized and the blood vessel density (BVD) quantified with micro-CT for intra-patient comparisons of native vaginal tissue, decellularized endodermal tissue and decellularized mesodermal tissue. The aim of this explorative study is to determine potential preservation of the vascular architecture in human-derived DVM, as this would allow future recellularization without need for neo-vascularization.

## Materials and methods

### Surgical procedure

Vaginal tissue from three healthy transgender patients (Appendix A1) was retrieved during colpectomy at Amsterdam UMC (the Netherlands) between January-December 2023^20^. Written informed consent for experimental use of resected vaginal tissue was obtained from all patients at least one month preoperatively. Their donation had no consequences for their psychological or medical transition. All tissue donors were presurgically exposed to androgen by testosterone injection (Nebido [Bayer Healthcare], Sustanon [Aspen]) or testosterone gel (Androgel [Besins Healthcare]). As no remarkable differences exist in the microvessel density of healthy genotypical males and females^41^, hormonal therapy is assumed to have no impact. Robotic-assisted laparoscopic colpectomy (daVinci XI system, Intuitive, Madrid, Spain) was performed^42^, as part of a patient’s medical transition. Vaginal epithelium was carefully dissected with monopolar scissors, and fenestrated bipolar forceps were used to minimize bleeding^43^. Vaginal tissue from epithelium, lamina propria and smooth muscle layers was removed in three rings, to maximize sample size without increasing surgical risks for the donor. Adventitia was preserved to prevent fistulas to bladder, urethra or rectum, nerve injury to adjacent structures, and bleeding from the perivaginal plexus^43^. The vaginal apex was laparoscopically closed with a suture. The surgically retrieved vaginal tissue was directly stored in phosphate buffered saline (PBS - Fresenius Kabi, Zeist, Utrecht, the Netherlands), pH 7.4, on ice for a maximum of 4 h until decellularization.

### Donated vagina tissue and chemical decellularization

Donated vaginal wall rings were researched anonymously. Any supplementary donor information was provided by the medical physician using coded sample description [VAG0##], the patient surgery date and Medical Dossier Number (MDN). Per patient three vaginal rings were retrieved, of which one mesodermal and one endodermal ring were chemically decellularized^20^, and a second mesodermal ring functioned as intrapatient native control. In brief, minimally required concentrations of Triton X-100, sodium deoxycholate and DNase I^44,45^ were applied for decellularization with minimal impact on vascular structures^46,47^. Exposure was limited to 24 h to minimize damage to the ECM structure and proteins^46^. Decellularization was initiated within 4 h, by 24 h incubation at 37 °C with constant agitation (100 motions/min) in 0.18% w/w Triton x-100 (Sigma-Aldrich, st. Louis, MO, USA) and 0.015% w/w sodium-deoxycholate (Sigma-Aldrich, st. Louis, MO, USA) in PBS^48^. Next, tissue was washed twice for 20 min in PBS, followed by 72 h at 4 °C with constant agitation in PBS with 1% PS (100 U/mL penicillin and 100 µg/mL streptomycin; Life Technologies Europe BV, Bleiswijk, Zuid-Holland, the Netherlands). Enzymatic digestion involved 24 h incubation at 37 °C with DNase I (150 IU/mL; Sigma-Aldrich, St. Louis, MO, USA) and 50 mmol MgCl_2_ (Sigma-Aldrich, st. Louis, MO, USA) in PBS. Lastly, 24 h incubation at 4 °C was performed with constant agitation in PBS with 1% PS, followed by an extensive PBS wash. The total protocol duration was 6 days. In our previous publication, successful decellularization was tested^20^ and confirmed through application of acellular criteria from in-vivo remodeling-reports^46^.

### Sample preparation microcomputed tomography

Vagina wall tissue (after decellularization) was fixed for 24 h in 4% paraformaldehyde (PFA) (w/v) in PBS (pH 7.4) at room temperature (RT). Tissue was stored at RT in a storage solution (0.2% PFA in PBS) until staining with 3.75% B-Lugol^49^ made from 7.5% Lugol’s solution (from 15% stock with 10 g KI and 5 g I_2_ dissolved in 100 mL bi-distilled water)^50,51^ and 2x Sorensen’s buffer (190.2 mM Na_2_HPO_4_ and 75.8 mM KH_2_PO_4_ to pH 7.2) in an 1:1 ratio. B-Lugol was used to enhance contrast of blood vessels while preventing typical soft-tissue shrinkage and associated artefacts when using unbuffered Lugol^52–55^. Tissue was stained a minimum of 24 h in B-Lugol at RT. Samples were rinsed with PBS for 3 h and mounted in 1.5% agarose in PBS to prevent movement artefacts during micro-CT scanning. This allowed non-invasive visualization of blood vessels and their branches, with a nominal resolution of 5 μm voxels (see Movie 1, 2 and 3).

### Microcomputed tomography with volumetric analysis and reconstruction

The 3D blood vessel micromorphology was visualized for native vagina wall tissue (only mesodermal tissue as positive control, since BVD of native mesoderm and endoderm vaginal tissue are equal^30^) and after chemical decellularization of endodermal and mesodermal vagina wall tissue. To correct for biological variation, blood vessel density was averaged over 11 volumes from three patients (*n* = 11). Tissue was imaged with a Phoenix Nanotom M (GE Sensing & Inspection Technologies GmbH, Germany) micro-CT at UC Louvain (Brussels, Belgium) with the following settings: X-ray tube voltage = 60 kV, X-ray tube current = 300 µA, exposure time = 500 ms, Filter = 0.2 Al, voxel size = 5 × 5 × 5 μm. The scan time depended on the field of view, which was determined with a rapid pre-scan and applied over all samples. Before segmentation, imaging of entire specimen was performed to assess spatial vessel density differences and to rule out anomalies. The images were imported and segmented semi-automatically in AMIRA 3D software (version 2021.2, Thermo Fisher Scientific, USA)^56^. A threshold intensity value was applied to prevent biased segmentation, and was manually checked and corrected for deviations. One large Volume of Interest (VOI) of 1 × 2.45 × 1.87 mm^3^ = 4.6 mm^3^ was analyzed per specimen for 3D animation of vascular structures. Quantitative analysis of vascular volume^57^ was performed with an additional 4.6 mm^3^ tissue from two other biological replicas, with 5 volumes per specimen of 1 × 0.49 × 0.935 mm^3^ = 0.46 mm^3^ each. The five VOIs were randomly selected for the first sample and evenly dispersed throughout the tissue volume, without displaying tissue features like blood vessels (blinded). The same VOI coordinates were used for sequential samples. Tissue volume, vascular volume, vascular length and vascular thickness were automatically computed from the segmented volumes. Next, the vascular density (total vasculature volume / total tissue volume*100%) and vascular connectivity (number of vasculatures / total tissue volume) were calculated.

### Statistical analysis

For 3D animation of vascular structures, weighted means were applied to correct for volumetric inconsistencies between biological replicas. Statistical analysis was performed on native mesodermal tissue (positive controls) and decellularized vaginal wall tissue of mesodermal and endodermal origin, to identify significant differences in density of vasculature. The weighted t-test was applied and results were expressed as mean with weighted standard deviation (WSD). Data were analyzed using the software Statistical Package for the Social Sciences version 28.0.1.1 for Windows (IBM Corp., Armonk, NY, USA). The p-value was obtained in a weighted, one-tailed paired t-test and was considered statistically significant for *p < 0.05, **p < 0.01 and ***p < 0.001.

### Method statement

The experiments were reported in accordance to STROBE guidelines.

### Ethical approvement statement

Tissue collection and experimental usage was approved by the institutional Medical Ethical Examination Committee of Amsterdam UMC location VU Medical Center (Amsterdam; IRB approval by METc VUmc registration number 2018/3190, October 2018) and performed according to the relevant guidelines and regulations.

## Results

### Reconstruction of vascular network in human native vagina and after decellularization

**Fig. 2:**
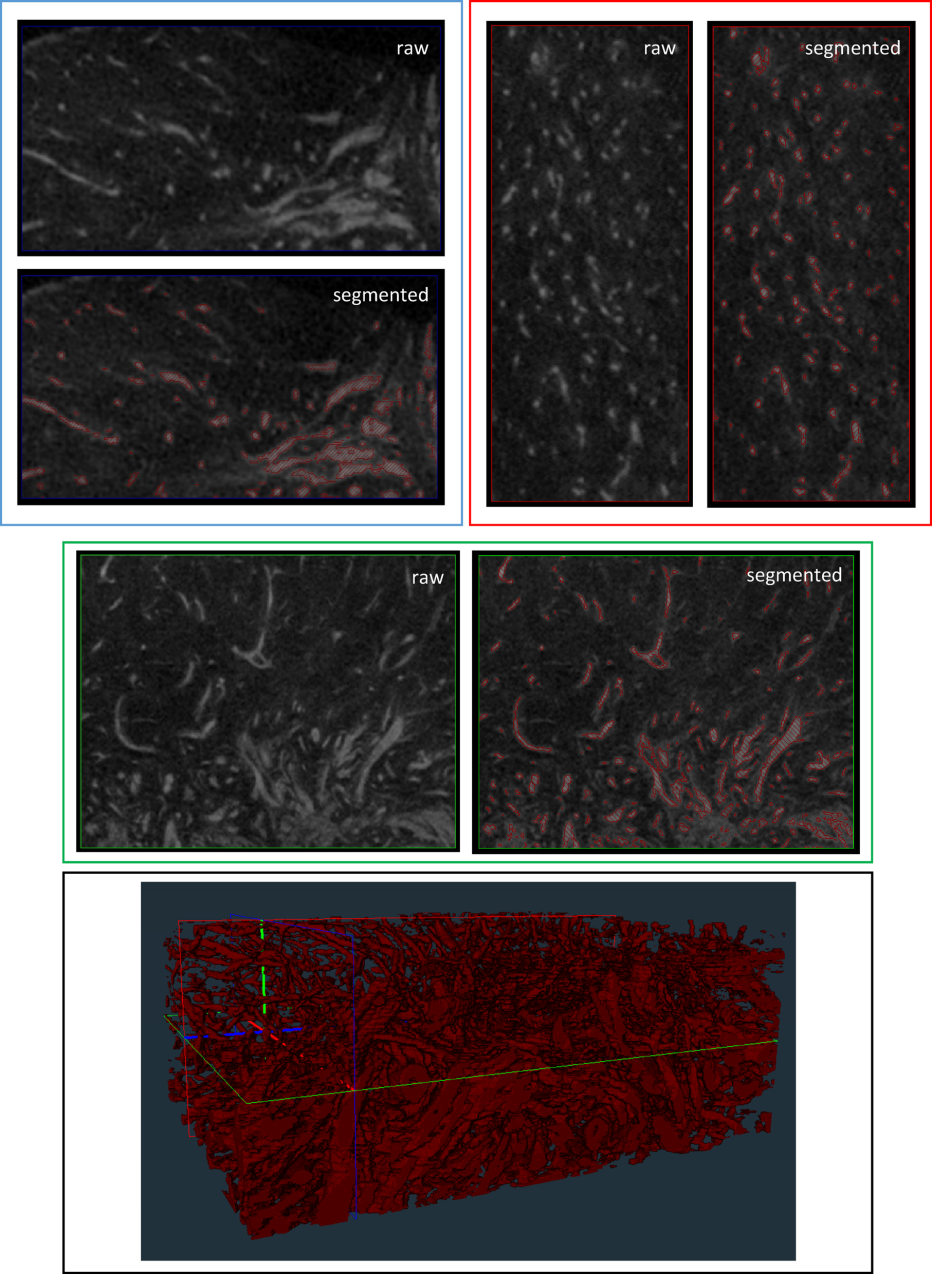
Manual segmentation process. Manual segmentation process for 3D reconstruction of microvascular networks in human vaginal wall from raw micro-CT data.

**Fig. 3:**
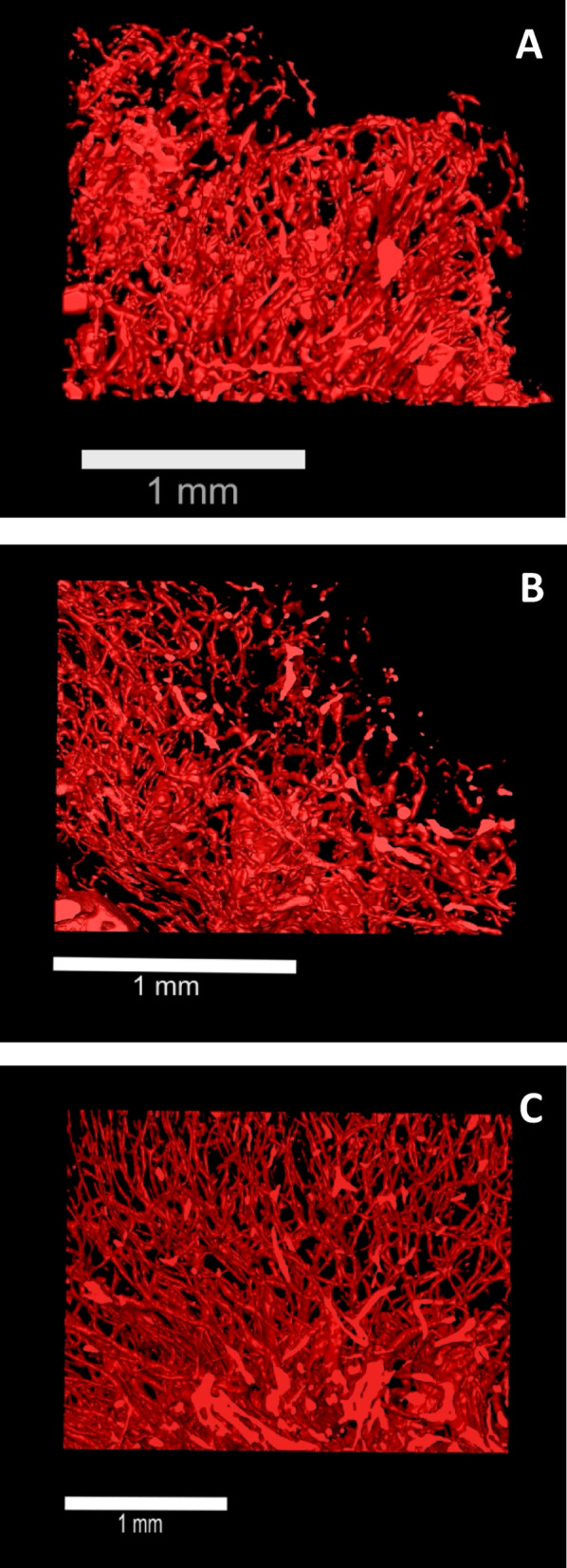
3D Reconstructive imaging of vasculature in human vaginal wall and vaginal matrix. 3D Reconstruction of vascular network in human vaginal wall by Micro-CT from manual segmentation of (**A**) native mesodermal tissue, (**B**) decellularized mesoderm sections and (**C**) decellularized endoderm sections. Reconstructions were performed for 3D animation of vascular structures on a large Volume of Interest (VOI) of 1 × 2.45 × 1.87 mm^3^ = 4.6 mm^3^ per specimen. Please note: scale bars vary in displayed size due to differences in VOI-orientations and maximization of the 3D reconstructed tissue to the image window.

Vagina wall tissue from three healthy transgender patients (Table S1) was included to reconstruct vascular networks for qualitative assessment. Vessels and their vascular branches can be distinguished from the micro-computed tomography (micro-CT) acquisition and were segmented for 3D reconstruction (Fig. 2). Depending on the plane of view, mostly cross or longitudinal sections of blood vessels can be observed. Vascular networks of native human vaginal wall (positive control) were distinguished and 3D reconstructed (Fig. 3A and Movie 1), as well as vascular features after chemical decellularization in human vaginal tissue of mesodermal origin (Fig. 3B and movie 2) and of endodermal origin (Fig. 3C and Movie 3). Visual representation of the analyzed Volume of Interests (VOIs) demonstrates that vascular structures in decellularized vaginal matrix of mesodermal and endodermal origin are comparable to that of native vaginal tissue, and seem to remain intact.

### Anatomical micro-CT imaging of human native vagina and after decellularization

**Fig. 4:**
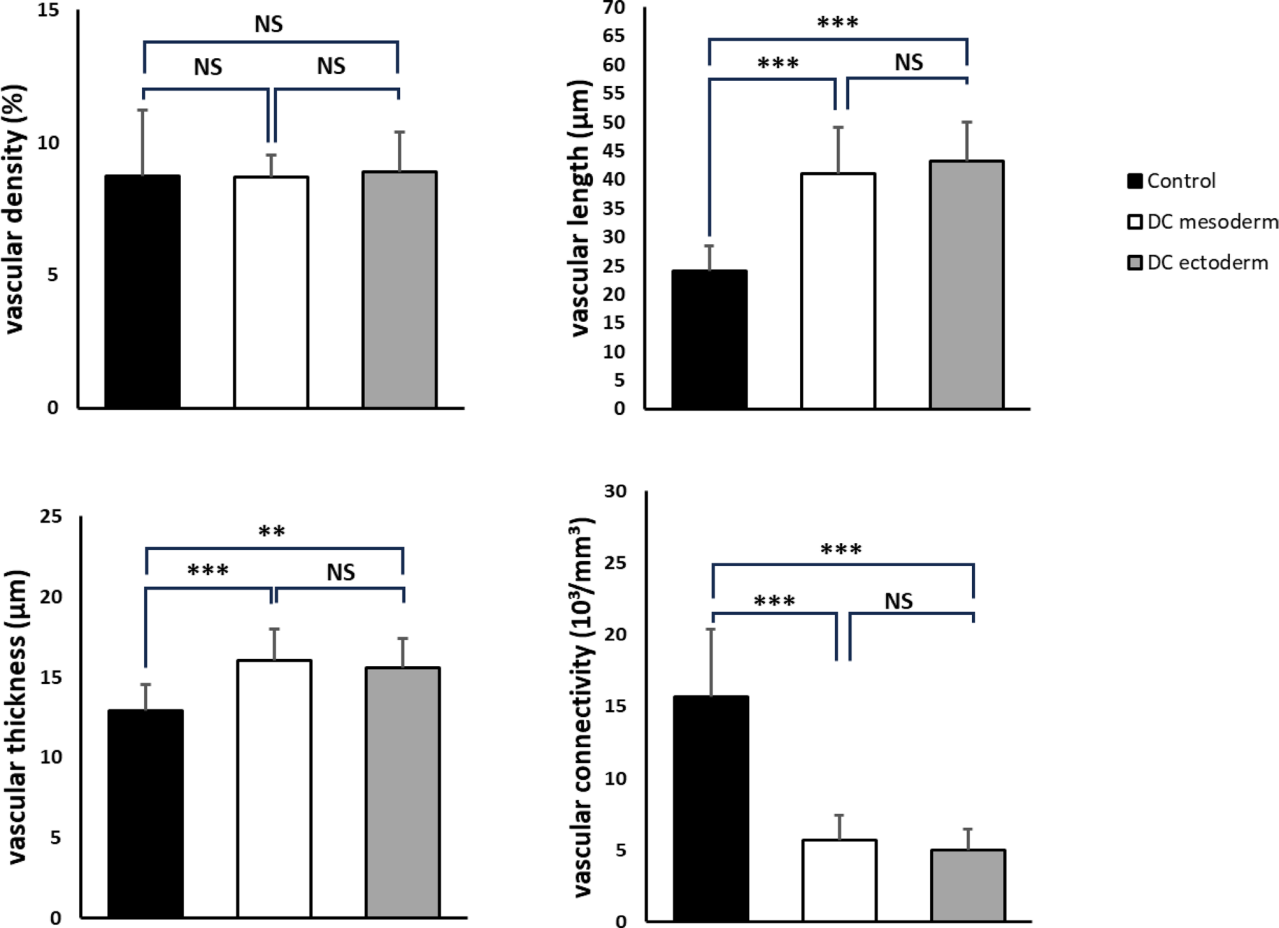
Quantification of vascular network density = artery volume/tissue volume (%), in native vagina (control), decellularized mesodermal vagina (DC mesoderm) and decellularized endodermal vagina (DC endoderm). Quantitative analysis was performed on a large 4.6 mm^3^ tissue segmentation for animation and 10 additional volumes from two other biological replicas. These additional volumes, with 5 volumes per biological replica, were of 1 × 0.49 × 0.935 mm^3^ = 0.46 mm^3^ in size each. Densities are not significantly (NS) decreased after decellularization. Significance is specified as *P < 0.05, **P < 0.01, and ***P < 0.001. For Control and DC mesoderm, *P* = 0.931. For Control and DC endoderm, *P* = 0.893. For DC mesoderm and DC endoderm, *P* = 0.721.

Micro-CT imaging was performed at a nominal resolution of 5 μm voxels. This is in theory sufficient to detect even small and newly formed blood vessels, that are typically smaller than 35 μm. Upon quantitative inspection of the microvasculature, blood vessels as small as 5 μm were observed. Density of vascular structures (Table 1; Fig. 4) in vaginal wall tissue (positive control) ranged from 2.9 to 44.9%, with a weighted average of 8.7 ± 2.5%. After decellularization, the vascular density was 2.0-19.2% with a weighted average of 8.7 ± 0.9% for mesoderm sections (*P* = 0.931), and a vascular density range of 1.6–23.5% with a weighted average of 8.9 ± 1.5% for endoderm sections (*P* = 0.893). The decellularized vagina sections did not significantly differ in vascular density (*P* = 0.721).

**Table 1:** Quantitative assessment of tissue volume, artery volume and artery density from vaginal tissue segmentation.

Sample	VOI (mm^3^)	Tissue volume (mm^3^)	Artery volume (mm^3^)	Artery density (%)
Weighted mean artery density = 8.75%;				
Control 1	1.87 × 2.45 × 1.0	2.02	0.17	8.49
Control 2	5* (1 × 0.49 × 0.94)	0.42	0.04	12.03
Control 3	5* (1 × 0.49 × 0.94)	0.44	0.03	5.97
Weighted mean artery density = 8.68%;				
DC mesoderm 1	1.87 × 2.45 × 1.0	2.40	0.23	9.39
DC mesoderm 2	5* (1 × 0.49 × 0.94)	0.42	0.03	8.26
DC mesoderm 3	5* (1 × 0.49 × 0.94)	0.40	0.03	7.69
Weighted mean artery density = 8.87%;				
DC ectoderm 1	1.87 × 2.45 × 1.0	4.08	0.38	9.39
DC ectoderm 2	5* (1 × 0.49 × 0.94)	0.42	0.04	10.07
DC ectoderm 3	5* (1 × 0.49 × 0.94)	0.44	0.03	6.65

Quantitative analysis was performed on a large 4.6 mm^3^ tissue segmentation for animation and 10 additional volumes from two other biological replicas. These additional volumes, with 5 volumes per biological replica, were of 1 × 0.49 × 0.935 mm^3^ = 0.46 mm^3^ in size each.

Based on visual and quantitative assessment, vascular networks of human vaginal wall tissue before and after decellularization demonstrate no density differences of statistical significance. A significant reduction of the vascular connectivity was seen from 15.7 × 10^3^ structures/mm^3^ in vaginal wall tissue (positive control) to 5.7 × 10^3^ structures/mm^3^ in decellularized mesoderm sections (*P* < 0.001) and to 5.0 × 10^3^ structures/mm^3^ in decellularized endoderm sections (*P* < 0.001). The average thickness per vascular structure increased significantly from 12.9 μm in vaginal wall tissue (positive control) to 16.1 μm in decellularized mesoderm sections (*P* < 0.001) and 15.6 μm in decellularized endoderm sections (*P* < 0.01). Furthermore, the average length per vascular structure increased significantly from 24.1 μm in vaginal wall tissue (positive control) to 41.1 μm in decellularized mesoderm sections (*P* < 0.001) and 43.3 μm in decellularized endoderm sections (*P* < 0.001).

## Discussion

### Key findings

This study performed imaging of 3D vascular structures to gain insight in potential preservation thereof in human decellularized vagina. We previously developed a human-derived DVM, which contained known (and perhaps also unknown or undiscovered) essential biomolecules with sufficient mechanical properties. However, its clinical success greatly relies on the presence of a tissue-engineered or preserved vasculature. This would allow recellularization, matrix integration and prevent surgical complications such as poor graft function and partial or complete graft loss. Although vascular-like structures were visible in DVM during previous histological assessment, vascular structures might not be (completely) preserved due to the removal of cells, and potentially collagen and elastin^20^. Therefore, in this explorative study we wanted to assess microvascular preservation by qualitative and quantitative analysis of the vascular architecture in native and decellularized vaginal tissue. Contrast-enhanced ex-vivo micro-CT was used for high-resolution visual analyses on 3D micromorphology of blood vessels. The anatomical information of native and decellularized vagina wall tissue was compared and both 3D reconstructions upon visual inspection demonstrated heterogenous blood vessel distribution with seemingly intact, mature vasculature. Mean blood vessel density of native and decellularized vaginal tissue were not significantly different, despite reduced connectivity with increased average thickness and length of vascular structures. This indicates significant loss of thin and short vascular structures, but with preservation of the larger microvascular architecture in endodermal and mesodermal DVM.

### Interpretations

Tissue vasculature is commonly investigated at high resolution by 2D-visualization, with limited scan area or by destructive methods (e.g. histology, immunofluorescence). However, 2D-imaging of vessels is limited in accuracy and is therefore an insufficient approach to study or diagnose angiogenesis^58^. Considerable research has utilized micro-CT for in -vivo imaging of anatomical anomalies and for assessment of vascular structures *in*/ex-vivo^32,33,35,37^^52^]–^55,59^. Yet, visualization of vasculature in tissue-engineered or decellularized constructs by micro-CT is sparse^60^. In this study, contrast-enhanced micro-CT was used to visualize the vasculature in native and decellularized vaginal tissue. With the applied scan conditions, a 5 μm isotropic voxel size resolution was achieved that is in theory sufficient to image microvessels (10–50 μm in size). Visual inspection confirmed that the micromorphology could be distinguished from its surrounding, and in-house resolutions of 1 μm voxels (not published) have been achieved with this setup at longer scan times, implicating that the system did not limit our results. The 3D reconstructions demonstrated a seemingly heterogenous distribution of the vascular architecture, which confirms previous reports on the vascular angioarchitecture of the vagina^61,62^. Observations of raw micro-CT scans did demonstrate differences in predominantly cross or longitudinal sections dependent on the selected 2D plane, thereby illustrating the importance of 3D over 2D study accuracy.

For this study, transgender patients donated vaginal wall tissue that would otherwise be disposed. Hence, this relatively complete vagina wall tissue offers a valuable and ethical resource. With respect to our intra-patient comparison of blood vessel density to assess the effect of decellularization, no indication exists for hormone-induced changes. Although vaginal atrophy^63^ is a reported hormone-induced effect on the vaginal vasculature, pathological findings confirmed the absence thereof in our samples (Table S1).

The blood vessel density (BVD = volume of blood vessels/volume of tissue) is a well-established quantitative indicator for neo-vascularization or preservation of vasculature, especially when used in combination with the average vascular thickness, length and connectivity^64–66^. The BVD has previously been combined with immunohistology and micro-CT^32,54,60^ and was applied here to demonstrate the 3D micromorphology of human vaginal wall and of decellularized vaginal matrix. There was no statistically significant difference between the BVDs, thereby suggesting that vascular architecture is preserved upon chemical decellularization. However, the number of vascular structures in the tissue volume (connectivity) reduced, while the average thickness and length increased at the same time. this indicates a major loss of short and thin capillaries. Furthermore, in combination with the absence of significant BVD changes, this implicates preservation of the large and longer vascular structures. Similar findings with respect to vascular preservation have previously been reported for decellularized liver^23,24^, heart^25,26^ and kidney^27,28^. The vasculature consists of endothelial cells with extracellular matrix and proteins such as laminin, collagen, elastin and hyaluronan^67^. Our previous study confirmed (mostly) retained laminin, collagen and elastin^20^, and supports our current findings. However, we want to emphasize that due to cell removal, preservation of vascular architecture in this context only refers to the vascular ECM. Lastly, with 5 μm voxels, no visual holes were detected in the vascular architecture.

### Implications

Compared to clinically applied imaging techniques for angiography and quantitative analysis of blood vessels (i.e. ultrasound, magnetic resonance imaging), micro-CT poses both advantages and disadvantages. Micro-CT is non-destructive, of low cost, with high resolution and high throughput, but requires X-ray exposure, and adjusted contrast enhancement. Furthermore, micro-CT requires either fast automated segmentation that is prone to errors in feature-dense, complex samples or manual segmentation that is more time-and labour-consuming^34^. In this study, we demonstrate that contrast enhanced micro-CT with 5 μm isotropic voxel size resolution is suitable for angiography of native and decellularized vaginal wall tissue. However, it can be applied to other native and tissue-engineered organs as well. Although in this study the blood vessel density, connectivity, average length and thickness were investigated, this 3D visualization method also allows quantification of parameters such as vessel distribution, orientation and branching. This information is especially relevant to study neo-vascularization or to identify tumorigenic angiogenesis^32^ for clinical use.

Beyond the impact of this micro-CT-based imaging method, the observed preservation of large-volume vasculature upon decellularization implies the potential of our decellularized vaginal tissue for clinical applications. Namely, preservation of the microvasculature or reconstruction of a neo-vasculature is essential for integration and survival upon implantation of a tissue-engineered human DVM. Although not part of this study, to ensure functionality of the preserved vascular ECM, we recommend future endothelization with host cells combined with animal-based validation experiments on long-term functional maintenance, blood flow capacity and absence of micro leaks. Ultimately, a complete and functional microvasculature in the DVM would allow for surgical connection to the patients’ blood supply and thereby guarantee graft survival.

### Limitations

This work is accompanied by some limitations. Validation of blood vessel density, connectivity, length and thickness across the full vaginal wall was not possible under current conditions, as time-consuming manual correction after semi-automated segmentation roughly required 240 h per animation or 10 h per quantitative sample assessment. This might create a slight bias for biological reproducibility and vessel density variation throughout the vaginal wall (for example: BVD range 2–19% for 5 VOIs within one sample), which could be resolved by completely automated segmentation of the entire sample combined with machine learning techniques to select resembling threshold intensities across samples. Although we used clear features to set thresholds, automated segmentation would strengthen the reproducibility of the presented methodology albeit at higher financial costs. This also relates to the use of specimen from 3 patients (biological replicas). The use of few biological replicas does not invalidate our study as this is common across similar publications of micro-CT analysis of vasculature in mice^53^, and DNA quantification from decellularized organ-derived scaffolds^24^. In this study, we completely scanned and assessed vagina walls, and used 11 sections for intra-patient quantitative comparison per condition. Therefore, we believe this data provides a valid indication of vascular network preservation in human vaginal wall tissue upon chemical decellularization. However, clinical applications and future studies could benefit from completely blinded image analysis, to prevent any risk of observer bias or inconsistencies. Furthermore, large-scale follow-up studies are required to strengthening the statistical power of this explorative study.

The small dimensions of vascular trees limit this study. Quantitative analysis ruled out large leaks or major destruction of vasculature. However, the current resolution cannot fully guarantee the absence of micro holes in the preserved parts of the vasculature. Furthermore, as our previous study showed that cells are completely removed from the vaginal matrix^20^, it may be assumed that only the ECM of the vasculature remained intact. In the current state, this vascular architecture is likely to contain holes at prior cell locations, therefore not providing a functional structure. Nonetheless, the significant intact part of vascular ECM can be repopulated with host cells (particularly endothelial cells, smooth muscle cells and fibroblasts) for its functionalization, and eliminates the need for complex neo-vascularization techniques^68^. In addition, the vascular ECM plays a pivotal role in maintaining and regulating vascular functions^69^. Lastly, the results from this study play a crucial role for recellularization, tissue survival and graft integration during clinical applicability, as the ECM vasculature (even with perforations) could allow distribution of oxygen, nutrient and cells beyond the diffusion limit. We are currently performing follow-up studies for repopulation of the vascular ECM, after which assessment of its perfusion capability (i.e., by blood flow and systolic/diastolic state) are recommended to validate full functioning of the DVM vasculature.

Lastly, this study is limited by the sole use of micro-CT. The sole use of micro-CT (in combination with histology) is similar to previous studies^28,37,54^ and we want to emphasize, as explained previously, that this is not a crucial aspect for the validity of our study. Furthermore, for 3D visualization of the vasculature without destruction of the sample, micro-CT is a state-of-the-art method that offers the required resolution while no suitable secondary control method is available. Ultrasound, magnetic resonance imaging and electron microscopy carry distinctive disadvantages for whole 3D tissue scans (e.g. limited penetration depth, low resolution, sample destruction). However, alternative information on the functionality of the vasculature could for instance be investigated through immunofluorescence microscopy or scanning electron microscopy. These are both part of our follow-up study that focusses on repopulation of our human DVM.

### Future perspective

Clinical applicability of bio-engineered vagina constructs relies on a viable matrix with suitable properties and the potential for repopulation with autologous cells. Our current study demonstrates that a human-derived DVM with mostly preserved vascular architecture is feasible. We are confident that our DVM therefore allows recolonization with host cells beyond the diffusion limit, which will facilitate tissue survival by recovery of any potential function loss of the vasculature. Furthermore, with the results from this study it is now possible to explore other potential challenges in the use of our DVM. Therefore, our follow-up studies address matrix sterility^70^, implant safety on cells and the use of autologous vaginal cells^71^ for (long-term) functionalization. Lastly, the applied micro-CT approach is in our opinion a feasible 3D application to (clinically) detect vaginal anomalies through distorted angiogenesis or blood vessel distribution. Thereto, machine-learning with complete automation is required, but the 3D reconstructions of this study can be used for initial assessment of AI-based approaches.

On a broader scale, we want to emphasize that this study was focused on the assessment of vascular structure preservation in DVM. To increase the generalizability of our findings, the results can potentially be used for cross-cohort and cross-study quantitative comparisons. For example, prior studies have reported a significant reduction of the total BVD in the vaginal wall due to low levels of estrogen at post-menopausal ages^72^. Large-scale follow-up of our study could be used to quantitatively assess the impact of duration-dependent, hormone-induced changes of the BVD. Similarly, large-scale studies with anomaly-free biopsies would allow investigation of hormone-induced BVD changes throughout pregnancy.

## Conclusion

For patients with a dysfunctional or absent vagina, various surgical procedures are available for neovagina creation. These methods all depend on adequate vascularization for graft functionality and survival. In this study, micro-CT was successfully implemented for 3D reconstruction with quantitative analysis of the blood vessel density, connectivity, average thickness and length in vaginal tissue, to obtain insight in vascular preservation in a human-derived decellularized vaginal matrix. Vascular ECM preservation was confirmed, thereby paving the way for endothelialization and colonization with host cells to recover vascular functions for follow-up in-vivo studies and clinical trials. Combined with previous findings, the vascular architecture, microstructure, structural proteins and mechanical properties of our human DVM are important predictors for its functionality. This study thereby provides an essential proof-of-principle for future surgical translation of our decellularized vaginal matrix, but also offers a quantification method for other tissue-engineered constructs that could be implemented prior to in-vivo studies to predict their functionality in terms of graft survival and integration.

## Supplementary Information

Below is the link to the electronic supplementary material.

Supplementary Material 1.

Supplementary Material 2.

Supplementary Material 3.

## Data Availability

Data are available from the corresponding author upon reasonable request.
