# Supplementary material for: Micro-computed tomography to visualize preserved vascular architecture in decellularized human vaginal tissue: explorative study
Source: Sci Rep. 2025 Aug 20;15:30533. doi: 10.1038/s41598-025-14452-8 (PMC12368158; doi:10.1038/s41598-025-14452-8)
Supplement: Supplementary file 2 — Supplementary Material 2. [file 41598_2025_14452_MOESM2_ESM.docx]

*Supplement Table I: Tissue donor characteristics.*

| Patient | Age (years) | Current Procedure | Pathological findings | Androgen Exposure (years) | ASA classification | Co-morbidity |
| --- | --- | --- | --- | --- | --- | --- |
| VAG013 | 24 | Total laporoscopic hysterectomy  bilateral salpingo-oophorectomy  Robot Colpectomy | NA | 6.0 | ASA2 | GD |
| VAG014 | 23 | Total laporoscopic hysterectomy  bilateral salpingo-oophorectomy  Robot Colpectomy | NA | 3.7 | ASA2 | GD |
| VAG015 | 46 | Total laporoscopic hysterectomy  bilateral salpingo-oophorectomy  Robot Colpectomy | NA | 1.0 | ASA2 | GD |
