# Supplementary material for: Micro-computed tomography to visualize preserved vascular architecture in decellularized human vaginal tissue: explorative study
Source: Sci Rep. 2025 Aug 20;15:30533. doi: 10.1038/s41598-025-14452-8 (PMC12368158; doi:10.1038/s41598-025-14452-8)
Supplement: Supplementary file 3 — Supplementary Material 3. [file 41598_2025_14452_MOESM3_ESM.docx]

Movie 1: VISUALIZATION OF RECONSTRUCTED 3D VASCULATURE IN HUMAN VAGINA WALL. Animation of 3D reconstructed vascular network in native vagina tissue. Total Volume of Interest (VOI) of 1 x 2.45 x 1.87 mm^3^ = 4.6 mm^3^. [Images should be hyperlinks to movies in published version, stills and movies in colour]

Movie 2: VISUALIZATION OF RECONSTRUCTED 3D VASCULATURE IN HUMAN DECELLULARIZED VAGINA WALL OF MESODERM ORIGIN. Animation of 3D reconstructed vascular network in decellularized mesodermal vagina tissue. Total Volume of Interest (VOI) of 1 x 2.45 x 1.87 mm^3^ = 4.6 mm^3^.

Movie 3: VISUALIZATION OF RECONSTRUCTED 3D VASCULATURE IN HUMAN DECELLULARIZED VAGINA WALL OF ENDODERM ORIGIN. Animation of 3D reconstructed vascular network in decellularized endodermal vagina tissue. Total Volume of Interest (VOI) of 1 x 2.45 x 1.87 mm^3^ = 4.6 mm^3^.
